# Impact of Personalized Recovery Interventions on Spinal Instability and Psychological Distress in Oncological Patients with Vertebral Metastases

**DOI:** 10.3390/diseases13030085

**Published:** 2025-03-16

**Authors:** Noémi Németh, Florica Voiță-Mekeres, Liviu Lazăr, Lavinia Davidescu, Călin Tudor Hozan

**Affiliations:** 1 Doctoral School of Biomedical Sciences, Faculty of Medicine and Pharmacy, University of Oradea, 410087 Oradea, Romania; nemeth.noemi@didactic.uoradea.ro (N.N.); lazarlv@yahoo.com (L.L.); 2 Department of Psycho-Neuroscience and Rehabilitation, University of Oradea, 410073 Oradea, Romania; 3 Department of Morphological Disciplines, Faculty of Medicine and Pharmacy, University of Oradea, 1 Universitatii Street, 410087 Oradea, Romania; 4 Department of Medical Disciplines, Faculty of Medicine and Pharmacy, University of Oradea, 1 Universitatii Street, 410087 Oradea, Romania; lavinia.davidescu@didactic.uoradea.ro; 5 Department of Surgical Disciplines, Faculty of Medicine and Pharmacy, University of Oradea, 410087 Oradea, Romania; chozan@uoradea.ro

**Keywords:** vertebral metastases, spinal instability neoplastic score (SINS), psychological distress, personalized recovery interventions, oncology

## Abstract

Background: Patients with vertebral metastases often experience spinal instability, chronic pain, and psychological distress, all of which can significantly reduce quality of life. Spinal instability, measured by the Spinal Instability Neoplastic Score (SINS), may exacerbate functional impairment and emotional distress, underscoring the potential benefit of personalized recovery interventions. Material and methods: This prospective, observational study investigated the impact of personalized recovery interventions on spinal instability, psychological distress, and quality of life in oncological patients with vertebral metastases. Results: The experimental group received tailored rehabilitation strategies, while the control group underwent standard oncological care. Spinal instability was assessed using the Spinal Instability Neoplastic Score (SINS), psychological distress was measured with the Hopelessness Depression Symptom Questionnaire (HDSQ), and quality of life was evaluated using the European Quality of Life-5 Dimensions (EQ-5D). The experimental group demonstrated significantly lower mean SINS scores, indicating reduced spinal instability, and lower HDSQ scores, suggesting decreased psychological distress. They also exhibited improvements in mobility, self-care, usual activities, and anxiety/depression dimensions of the EQ-5D. Furthermore, the experimental group had longer survival times, lower fracture rates, and reduced prevalence of osteoporosis, anemia, and vomiting. These findings underscore the potential benefits of integrating physical and psychological rehabilitation into routine oncological management. Conclusions: Personalized recovery interventions appear to enhance functional independence, emotional well-being, and overall quality of life in patients with vertebral metastases. Future research should focus on longitudinal, multicenter, randomized controlled trials to confirm these findings and further elucidate the complex interplay between spinal instability, psychological distress, and functional recovery.

## 1. Introduction

Cancer remains a major global public health challenge, with an increasing number of survivors due to advances in early diagnosis, treatment, and demographic shifts such as population aging [1,2]. Cancer treatments, including surgery, radiotherapy, and chemotherapy are essential for disease control but often contribute to substantial physical and psychological burdens. Chemotherapy, in particular, is associated with a range of side effects that can significantly diminish quality of life (QoL), including fatigue, sleep disturbances, nausea, cognitive impairment, and psychological distress [3,4]. Anxiety and depression are among the most prevalent mental health conditions in oncology, affecting up to 20% of cancer patients, with implications for disease progression, treatment adherence, and overall survival [5,6].

Among cancer patients with vertebral metastases, pain and disability related to spinal instability can further compound psychological distress. The Spinal Instability Neoplastic Score (SINS) was introduced by the Spine Oncology Study Group in 2010 to assess the degree of spinal instability in metastatic disease, guiding decisions regarding surgical intervention [7,8]. The score incorporates both radiographic and clinical parameters, classifying patients as having stable, potentially unstable, or unstable spines. Although SINS has been widely used in oncological practice, its relationship with mental health outcomes and QoL remains largely unexplored. Given that spinal instability often leads to chronic pain, functional limitations, and reduced mobility, understanding its association with depression and psychological distress is critical for optimizing patient care [9,10].

Psychological distress in cancer patients is multifactorial, influenced by disease burden, treatment side effects, and sociodemographic factors [9]. Depression, a mood disorder characterized by persistent sadness, hopelessness, and anhedonia, has been linked to longer hospital stays, poorer tolerance to chemotherapy, and reduced QoL [11,12]. Despite the well-established prevalence of depression and anxiety in oncology, these conditions remain underdiagnosed and undertreated, limiting opportunities for timely intervention [13,14]. Understanding how spinal instability, functional impairment, and depression interact could provide critical insights into tailoring multidisciplinary rehabilitation strategies.

This study aims to investigate the impact of spinal instability (as measured by SINS), depressive symptoms, and QoL in oncological patients with vertebral metastases. We hypothesize that higher SINS scores will be associated with increased psychological distress and lower QoL due to their influence on pain severity and functional impairment. By identifying these associations, our findings could support the development of personalized recovery interventions that integrate both physical and psychological rehabilitation for this vulnerable patient population.

## 2. Materials and Methods

### 2.1. Study Population

This prospective, observational study was conducted over a two-year period (2022–2024) to assess the impact of personalized recovery interventions on oncological patients with vertebral metastases. The study aimed to evaluate functional independence and psychological distress in response to structured rehabilitation strategies. A comparative approach was used, where an experimental group received personalized interventions, while a control group followed standard oncological care.

The study included adult patients aged 18 years or older who were newly diagnosed or currently undergoing oncological treatment. Eligibility required radiological confirmation of vertebral metastases and the presence of spinal pain (rachialgia). Participants had to provide informed consent and demonstrate preserved cognitive function to ensure reliable self-reported assessments. Exclusion criteria encompassed pediatric patients, those without radiological confirmation of vertebral metastases, individuals with neuropsychiatric disorders that impaired judgment, and patients who died during the selection period. Individuals with severe functional limitations that precluded participation in rehabilitation interventions were also excluded.

### 2.2. Instruments

Psychological distress was assessed using the Hopelessness Depression Symptom Questionnaire (HDSQ), which measures symptoms of hopelessness and depression through 32 items grouped into eight subscales: motivational deficit, interpersonal dependency, psychomotor retardation, lack of energy, apathy/anhedonia, insomnia, concentration difficulty, and suicidality. Each subscale consists of four items, with responses aggregated into total and domain-specific scores. Metalsky and Joiner (1997) [15] reported Cronbach’s alpha coefficients ranging from 0.70 to 0.93, confirming the scale’s reliability. In this study, the HDSQ demonstrated high internal consistency, with a Cronbach’s alpha of 0.91.

Spinal instability was assessed using the Spinal Instability Neoplastic Score (SINS), which evaluates tumor location, pain severity, bone lesion type (lytic, blastic, or mixed), spinal alignment, vertebral body collapse, and posterolateral involvement of spinal elements such as pedicles and facets. The total score categorizes spinal stability into three groups: stable (0–6 points), potentially unstable (7–12 points), and unstable (13–18 points).

Quality of life was assessed using the European Quality of Life-5 Dimensions (EQ-5D), a widely used measure that evaluates mobility, self-care, usual activities, pain/discomfort, and anxiety/depression. Scores were analyzed both as individual domain scores and as an overall index score. Additionally, the Spinal Oncology Study Group Outcomes Questionnaire (SOSGOQ-2.0) was used to assess spine-specific QoL parameters, including pain severity, physical function, social participation, and mental well-being.

### 2.3. Intervention and Ethical Consideration

Patients in the experimental group received personalized recovery interventions that were tailored to their mental and functional reserves, vertebral fracture risk, physical endurance, and fatigue levels, while considering their ongoing oncological therapies, which could include chemotherapy, radiotherapy, or surgical interventions. The rehabilitation program integrated physiotherapy and functional rehabilitation focused on postural correction, mobility training, and muscle strengthening. Psychological support was provided through structured counseling and cognitive-behavioral therapy (CBT) to manage anxiety, depression, and pain perception, and nutritional guidance was offered to optimize dietary intake for bone health and energy levels. Pain management strategies included medication adjustments, nerve blocks, and non-pharmacological approaches such as mindfulness-based stress reduction techniques. The study was conducted in accordance with the Declaration of Helsinki, and all participants provided written informed consent. Ethical approval was obtained from the Institutional Ethics Committee (reference numbers 42571/16.12.2022), with strict adherence to confidentiality and data protection protocols throughout the study.

### 2.4. Statistical Methods

Statistical analyses were performed to evaluate the impact of the interventions on functional independence and psychological distress. Data underwent verification for accuracy and completeness, with appropriate methods used for handling missing values. Continuous variables were summarized using means, standard deviations, medians, and ranges, while categorical variables were reported as frequencies and percentages. The normality of continuous variables was assessed using the Shapiro–Wilk test. For normally distributed data, independent t-tests were used to compare means between the experimental and control groups, whereas non-parametric alternatives such as the Mann–Whitney U test were applied for skewed data. Categorical data were analyzed using chi-square tests, and Fisher’s exact test was employed when expected cell counts were less than five.

Quality of life measures were analyzed using ANOVA for multiple group comparisons and t-tests for two-group comparisons. To control the risk of Type I errors, multiple testing corrections such as the Bonferroni adjustment were applied. Factor analysis was conducted to validate psychometric scales.

In addition to significance testing, effect sizes were calculated to quantify the magnitude of differences between groups. Specifically, Hedges’ g was employed to adjust for small sample bias, providing a more accurate measure of effect size when sample sizes are moderate or unequal. As highlighted by Durlak (2009) [16], reporting effect sizes alongside *p*-values offers a clearer insight into the practical significance of the findings. Hedges’ g values were interpreted using conventional benchmarks, where values of approximately 0.2, 0.5, and 0.8 are indicative of small, medium, and large effects, respectively.

All statistical analyses were conducted using R, version 4.4.2. The statistical workflow incorporated the stats package for normality testing, t-tests, and ANOVA; the psych package for descriptive statistics and factor analysis; the coin package for Mann–Whitney U tests; and the Exact 2 × 2 package for Fisher’s exact tests. Multiple testing corrections were implemented using the Multcomp package or the p.adjust function. All statistical tests were two-tailed, with significance set at *p* < 0.05.

## 3. Results

The demographic and baseline clinical characteristics of the experimental and control groups were compared, and the findings reveal both similarities and notable differences between the groups.

The gender distribution showed no significant difference between the experimental and control groups (*p* = 0.766), with females comprising approximately 64.6% of the total sample and males representing 35.4%. Similarly, there were no significant differences in age (*p* = 0.858), with the mean age being 64.3 years (SD = 11.9) across both groups, ranging from 27 to 89 years.

The environmental distribution of participants (urban vs. rural) also did not differ significantly between groups (*p* = 0.885). The urban population constituted 59.1% of the total cohort, while the rural population accounted for 40.9%. Regarding education, both groups had a balanced distribution across educational levels, and no significant differences were noted (*p* = 0.863). Most participants had secondary education (29.8%) or vocational training (26.8%).

However, marital status differed significantly between the groups (*p* = 0.014). The experimental group had a higher proportion of married individuals (62.6%) compared to the control group (48.5%), while the control group had a larger percentage of unmarried and cohabiting participants. Widowed and divorced proportions were relatively similar between groups.

For diabetes, a statistically significant difference was observed (*p* = 0.006), with a higher prevalence of diabetes in the control group (52.5%) compared to the experimental group (33.3%). In contrast, there was no significant difference in the distribution of hypertension (HTA) grades between groups (*p* = 0.338), with most participants classified as Grade 2 (46.0%).

Lastly, smoking status was evenly distributed between the groups, with no significant difference (*p* = 1.000); 50.5% of participants in both groups were non-smokers.

While the groups were generally well-matched in terms of sex, age, environment, education, hypertension grade, and smoking status, significant differences in marital status, diabetes prevalence, and BMI highlight potential areas requiring adjustment or consideration in subsequent analyses. These findings underscore the importance of interpreting outcomes in the context of baseline variability (Table 1).

The analysis of clinical and radiological characteristics revealed notable differences and similarities between the experimental and control groups.

Tumor location was uniformly distributed across both groups, with 91.9% of participants presenting with multiple locations, and no significant difference was observed (*p* = 1.000). Similarly, for pain severity, there were no statistically significant differences between groups (*p* = 0.081). However, severe pain was reported more frequently in the control group (77.8%) compared to the experimental group (64.6%).

The type of bone lesion distribution was nearly identical between groups, with lytic lesions being observed in 33.3% of the experimental group and 35.4% of the control group, and blastic lesions being the most common overall (58.6%, *p* = 0.993). Mixed lesions were rare (1.0% in both groups).

In terms of spinal alignment, most participants in both groups exhibited moderate deformation (68.7% in the experimental group and 74.7% in the control group), with no significant difference between groups (*p* = 0.286). Severe deformations were slightly more frequent in the control group (15.2%) compared to the experimental group (13.1%).

A significant difference was noted in vertebral body collapse (*p* = 0.006). No collapse cases were more common in the experimental group (38.4%) compared to the control group (16.2%), while partial collapse was more frequent in the control group (68.7%) than in the experimental group (50.5%). Severe collapse was observed in 12.1% of the control group versus 9.1% of the experimental group.

Finally, the extent of posterolateral involvement of spinal elements (pedicles and facets) did not differ significantly between groups (*p* = 0.170). Mild involvement was the most common in both groups (45.5% in the experimental group and 46.5% in the control group), while severe involvement was rare, occurring in only one participant in the experimental group (1.0%) (Table 2).

For the Spinal Instability Neoplastic Score (SINS), the mean score was significantly lower in the experimental group (9.6 ± 2.8) compared to the control group (10.5 ± 2.4, *p* = 0.018). While both groups demonstrated a similar range (3.0–16.0 in the experimental group and 5.0–16.0 in the control group), the difference in means suggests variations in the extent of spinal instability (Table 3).

The analysis of treatment strategies between the experimental and control groups highlights distinct patterns in the use of radiotherapy, chemotherapy, and surgical interventions.

For radiotherapy, a statistically significant difference was observed between the two groups (*p* < 0.001). A substantially higher percentage of patients in the experimental group (94.9%) received radiotherapy compared to the control group (70.7%). Conversely, the control group had a significantly higher proportion of patients who did not receive radiotherapy (29.3%) compared to the experimental group (5.1%).

In terms of chemotherapy, no significant differences were noted between the two groups (*p* = 0.838). Most patients in both groups (74.7%) received a standard chemotherapy regimen, while intensive chemotherapy was rare, reported in only 2.0% of the experimental group and 1.0% of the control group. A similar proportion of patients in both groups (23.2% in the experimental group and 24.2% in the control group) did not receive chemotherapy.

For surgical treatment, significant differences were observed (*p* = 0.022). A higher percentage of patients in the control group underwent surgery (39.8%) compared to the experimental group (24.5%). Conversely, the experimental group had a greater proportion of patients who did not undergo surgery (75.5%) compared to the control group (60.2%). Missing data regarding surgical status were minimal (1.0% in both groups) (Table 4).

The analysis of survival time and complications between the experimental and control groups demonstrates notable differences in clinical outcomes. Survival time from diagnosis was significantly longer in the experimental group, with a mean of 3.5 years (SD = 1.3) compared to 2.8 years (SD = 1.1) in the control group. The range of survival time extended from 0.0 to 7.0 years in the experimental group and from 0.0 to 5.0 years in the control group. This difference was statistically significant, with a *p*-value of less than 0.001, indicating a clear advantage in survival for the experimental group.

The prevalence of complications also varied significantly between the groups. Fractures were observed in 20.4% of patients in the experimental group, compared to 56.6% in the control group. Most patients in the experimental group (79.6%) did not experience fractures, while only 43.4% of control group patients remained fracture-free. This difference, with a *p*-value of less than 0.001, highlights a marked reduction in fracture rates in the experimental group. Similarly, osteoporosis was present in 17.3% of patients in the experimental group and 37.8% in the control group, with a significant *p*-value of 0.001. The absence of osteoporosis was documented in 82.7% of patients in the experimental group and 62.2% in the control group, further emphasizing the protective effect observed in the experimental group.

Anemia was significantly less prevalent in the experimental group, affecting 22.7% of patients compared to 56.6% in the control group, with a *p*-value of less than 0.001. Most patients in the experimental group (77.3%) did not develop anemia, in contrast to only 43.4% of patients in the control group. Vomiting was another complication with a significant disparity between groups. It occurred in only 6.2% of the experimental group, compared to 53.5% of the control group. This difference, with a *p*-value of less than 0.001, underscores a notable reduction in vomiting in the experimental group.

Pathological fractures showed additional variations. General pathological fractures were more frequent in the control group, with a mean fracture score of 0.6 (SD = 0.5) compared to 0.2 (SD = 0.4) in the experimental group. This difference was statistically significant, with a *p*-value of less than 0.001. Spinal fractures were also less common in the experimental group, affecting 18.2% of patients compared to 32.3% in the control group, with a significant *p*-value of 0.022. However, differences in upper limb fractures and lower limb fractures between the groups were not statistically significant, with *p*-values of 0.070 and 0.074, respectively (Table 5).

The comparison of dimensions between the experimental and control groups highlights distinct trends across various categories, emphasizing both significant and non-significant differences.

In the mobility dimension, significant differences were observed between the groups, with a *p*-value of less than 0.001. In the experimental group, 14.1% of patients reported minimal mobility limitations, compared to none in the control group. Moderate mobility issues were reported by 55.6% of the experimental group and 49.5% of the control group. Severe mobility issues were more frequent in the control group, affecting 50.5% of patients compared to 30.3% in the experimental group, demonstrating an overall better mobility profile for the experimental group.

The self-care dimension also revealed significant differences, with a *p*-value of 0.006. A higher percentage of patients in the experimental group (12.1%) reported minimal self-care limitations, compared to only 1.0% in the control group. Moderate self-care limitations were comparable between the groups, affecting 62.6% of the experimental group and 67.7% of the control group. Severe limitations (category 3) were slightly less prevalent in the experimental group (25.3%) compared to the control group (31.3%).

For usual activities, significant differences were noted with a *p*-value of 0.004. Minimal interference with usual activities (category 1) was reported by 4.0% of the experimental group and none of the control group. Moderate limitations were more common in the experimental group (72.7%) than in the control group (57.6%). Severe limitations were more prevalent in the control group, affecting 42.4% of patients compared to 23.2% in the experimental group.

The pain/discomfort dimension did not reveal statistically significant differences between the groups, with a *p*-value of 0.446. Minimal pain (category 1) was reported by 1.0% of the experimental group, while no patients in the control group fell into this category. Moderate pain affected 48.5% of the experimental group and 43.4% of the control group. Severe pain was more common, affecting 50.5% of the experimental group and 56.6% of the control group.

In the anxiety/depression dimension, significant differences were observed, with a *p*-value of less than 0.001. Minimal anxiety/depression (category 1) was reported by 10.1% of the experimental group, while none of the control group reported this level. Moderate anxiety/depression was more frequent in the experimental group (77.8%) compared to the control group (59.6%). Severe anxiety/depression was substantially higher in the control group, affecting 40.4% of patients compared to 12.1% in the experimental group (Table 6).

The analysis of the Helplessness Depression Symptom Questionnaire (HDSQ) scores highlights significant differences between the experimental and control groups, as indicated by a *p*-value of less than 0.001.

The mean HDSQ score was significantly lower in the experimental group, with a value of 52.1 (SD = 17.8), compared to 63.5 (SD = 12.9) in the control group. This suggests that participants in the experimental group experienced lower levels of helplessness and depressive symptoms compared to those in the control group. The overall mean score across both groups was 57.8 (SD = 16.5).

Regarding the range of scores, the experimental group displayed a broader distribution, ranging from 23.0 to 96.0, while the control group scores ranged from 38.0 to 96.0. This wider range in the experimental group may indicate a more heterogeneous response to the intervention.

In conclusion, the significantly lower mean HDSQ score in the experimental group suggests that the intervention was associated with reduced symptoms of helplessness and depression compared to the control group. These findings underscore the potential efficacy of the intervention in alleviating psychological distress among participants (Table 7).

## 4. Discussion

This study evaluated the impact of spinal instability, psychological distress, and quality of life (QoL) of oncological patients with vertebral metastases, with a specific focus on how personalized recovery interventions influence these outcomes. Our findings demonstrate that patients receiving personalized rehabilitation exhibited significantly lower levels of psychological distress, enhanced mobility, and improved QoL compared to those undergoing standard care. These results underscore the potential benefits of integrating both physical and psychological rehabilitation into routine oncological management.

A key observation was the association between higher Spinal Instability Neoplastic Score (SINS) values and increased psychological distress. Patients with greater spinal instability reported higher levels of anxiety, depression, and functional impairment. This association persisted even after accounting for pain severity, suggesting that spinal instability may contribute to a decline in mental health. In the experimental group, personalized recovery interventions not only improved psychological well-being but were also linked with lower fracture rates and reduced prevalence of osteoporosis, further supporting the role of comprehensive rehabilitation strategies.

Our findings are consistent with, yet extend, previous research. Earlier studies by Hussain et al. (2018, 2019) [17,18] reported only weak correlations between SINS and general pain measures, such as those assessed by the Brief Pain Inventory (BPI) and the MD Anderson Symptom Inventory—Spine Tumor (MDASI) questionnaire. This discrepancy may be attributed to the spine-specific focus of our measurement tools, which more precisely capture the impact of spinal pain on function and mental health. Additionally, previous studies have highlighted the significant impact of spinal metastases on overall functionality and pain levels, yet few have explored their direct effects on psychological distress and QoL [19,20,21].

Furthermore, while previous research has established the high prevalence of anxiety and depression among cancer patients (Mitchell et al., 2011; Wang et al., 2020) [13,14], few studies have examined the influence of spinal instability on these conditions. Our results demonstrate that higher SINS scores are associated with poorer mental health outcomes, independent of pain severity. The bidirectional relationship between spinal instability, mental health, and QoL is particularly noteworthy. Patients with severe spinal instability may limit physical activity due to fear of fractures or worsening pain, leading to further functional decline and an exacerbation of psychological distress. Persistent pain can also disrupt cognitive function, sleep quality, and emotional regulation, compounding the overall burden of spinal instability (Charalambous et al., 2017) [22]. These findings align with those reported in the literature that spinal instability in metastatic cancer patients significantly influenced their psychological resilience, further contributing to a decline in emotional well-being and coping mechanisms [23,24].

In addition, evidence from previous studies indicates that higher SINS scores correlate with worse pain and disability measures. For example, Hussain et al. (2017) [17] reported significant positive correlations between increasing SINS and the severity of preoperative pain and disability, while Pennington et al. (2019) [25] noted that lesions with SINS ≥ 10 had a more than 50% probability of undergoing stabilization, compared to only 11% for patients with SINS ≤ 9. These findings suggest that a threshold around SINS 9–10 may mark a critical point at which biomechanical instability becomes more clinically significant, affecting both physical and psychological outcomes. Similarly, Versteeg et al. (2021) [26] found that early intervention in patients with high SINS scores led to better functional outcomes and reduced rates of severe disability. Moreover, a systematic review by Serratrice et al. (2022) emphasized the role of early surgical intervention in limiting the physical and psychological burden of vertebral metastases, highlighting the importance of early assessment and treatment [27].

The clinical implications of our findings are substantial. Personalized recovery interventions that combine physical therapy, pain management, and psychological counseling appear to enhance functional independence and emotional well-being, potentially reducing the overall burden of vertebral metastases. Moreover, the observed improvements in mobility, along with the reductions in fracture and osteoporosis rates, suggest that early rehabilitation may have a preventive role and could contribute to prolonged survival. A meta-analysis by Hamash and Walker (2023) [28] supports the effectiveness of multidisciplinary rehabilitation programs in improving pain management and QoL in cancer patients with bone metastases. This is also consistent with the broader literature on cancer rehabilitation, where integrated approaches have been shown to reduce psychological distress and enhance overall health outcomes [29,30,31,32].

Despite these promising results, there are several limitations that must be acknowledged. The relatively small sample size may limit the general applicability of our findings across diverse oncological populations. The cross-sectional design restricts our ability to infer causality between spinal instability, psychological distress, and QoL, and unmeasured factors—such as prior psychiatric history and opioid usage—may have influenced the outcomes. Additionally, although SINS is a validated tool for assessing spinal instability, it does not capture dynamic biomechanical factors. Future studies employing imaging-based biomechanical assessments, such as dynamic MRI or finite element analysis, may provide greater precision in evaluating instability.

This study provides robust evidence that personalized recovery interventions can significantly improve mental health, physical function, and overall QoL in patients with vertebral metastases. The integration of physical and psychological rehabilitation strategies into standard oncology care may markedly enhance patient outcomes. Future research should focus on longitudinal, multicenter, randomized controlled trials to confirm these findings and further elucidate the complex interplay between spinal instability, psychological distress, and functional recovery. Expanding research to include different oncological populations with varied metastasis locations could also provide further insights into how spinal instability interacts with broader systemic cancer effects.

## 5. Conclusions

This study demonstrates that personalized recovery interventions significantly reduce spinal instability and psychological distress while enhancing quality of life in oncological patients with vertebral metastases. By integrating targeted physiotherapy, pain management, and psychological support, these interventions not only improve functional independence, but also lower complication rates and extend survival. Future longitudinal, multicenter studies are warranted to further validate these findings and clarify the underlying mechanisms.

## Figures and Tables

**Table 1:** Comparison of Demographic and Clinical Characteristics Between Experimental and Control Groups.

Variable	Experimental (N = 99)	Control (N = 99)	Total (N = 198)	*p*-Value
Sex				0.766
- Female	63 (63.6%)	65 (65.7%)	128 (64.6%)	
- Male	36 (36.4%)	34 (34.3%)	70 (35.4%)	
Age				0.858
- Mean (SD)	64.5 (11.9)	64.2 (11.9)	64.3 (11.9)	
- Range	27–89	27–88	27–89	
Environment				0.885
- Rural	41 (41.4%)	40 (40.4%)	81 (40.9%)	
- Urban	58 (58.6%)	59 (59.6%)	117 (59.1%)	
Education				0.863
- Gymnasium	13 (13.1%)	14 (14.1%)	27 (13.6%)	
- Vocational school	28 (28.3%)	25 (25.3%)	53 (26.8%)	
- High school	29 (29.3%)	30 (30.3%)	59 (29.8%)	
- Higher education	28 (28.3%)	30 (30.3%)	58 (29.3%)	
- Other	1 (1.0%)	0 (0.0%)	1 (0.5%)	
Marital Status				0.014
- Unmarried	1 (1.0%)	6 (6.1%)	7 (3.5%)	
- Married	62 (62.6%)	48 (48.5%)	110 (55.6%)	
- Divorced	11 (11.1%)	17 (17.2%)	28 (14.1%)	
- Cohabitation	2 (2.0%)	10 (10.1%)	12 (6.1%)	
- Widowed	23 (23.2%)	18 (18.2%)	41 (20.7%)	
Diabetes				0.006
- No	66 (66.7%)	47 (47.5%)	113 (57.1%)	
- Yes	33 (33.3%)	52 (52.5%)	85 (42.9%)	
HTA Grade				0.338
- No HTA	17 (17.2%)	15 (15.2%)	32 (16.2%)	
- Grade 1	32 (32.3%)	42 (42.4%)	74 (37.4%)	
- Grade 2	50 (50.5%)	41 (41.4%)	91 (46.0%)	
- Grade 3	0 (0.0%)	1 (1.0%)	1 (0.5%)	
BMI				0.027
- Mean (SD)	21.2 (4.4)	22.7 (5.4)	21.9 (4.9)	
- Range	2.5–38.9	17.3–38.9	2.5–38.9	
Smoking				1.000
- No	50 (50.5%)	50 (50.5%)	100 (50.5%)	
- Yes	49 (49.5%)	49 (49.5%)	98 (49.5%)	

**Table 2:** Analysis of Clinical and Radiological Characteristics in Experimental and Control Groups.

Variable	Experimental (N = 99)	Control (N = 99)	Total (N = 198)	*p*-Value
Tumor Location				1
- Single Location	8 (8.1%)	8 (8.1%)	16 (8.1%)	
- Multiple Locations	91 (91.9%)	91 (91.9%)	182 (91.9%)	
Pain (Mechanical, instability-related, aggravated by movement)				0.081
- No Pain	1 (1.0%)	0 (0.0%)	1 (0.5%)	
- Mild	31 (31.3%)	22 (22.2%)	53 (26.8%)	
- Moderate	3 (3.0%)	0 (0.0%)	3 (1.5%)	
- Severe	64 (64.6%)	77 (77.8%)	141 (71.2%)	
Type of Bone Lesion				0.993
- No Lesion	6 (6.1%)	6 (6.1%)	12 (6.1%)	
- Lytic	33 (33.3%)	35 (35.4%)	68 (34.3%)	
- Blastic	59 (59.6%)	57 (57.6%)	116 (58.6%)	
- Mixed	1 (1.0%)	1 (1.0%)	2 (1.0%)	
Spinal Alignment (Deformation due to tumor)				0.286
- Normal	16 (16.2%)	10 (10.1%)	26 (13.1%)	
- Mild Deformation	2 (2.0%)	0 (0.0%)	2 (1.0%)	
- Moderate Deformation	68 (68.7%)	74 (74.7%)	142 (71.7%)	
- Severe Deformation	13 (13.1%)	15 (15.2%)	28 (14.1%)	
Vertebral Body Collapse				0.006
- No Collapse	38 (38.4%)	16 (16.2%)	54 (27.3%)	
- Partial Collapse	50 (50.5%)	68 (68.7%)	118 (59.6%)	
- Moderate Collapse	2 (2.0%)	3 (3.0%)	5 (2.5%)	
- Severe Collapse	9 (9.1%)	12 (12.1%)	21 (10.6%)	
Posterolateral Involvement of Spinal Elements (Pedicles, Facets)				0.17
- No Involvement	25 (25.3%)	15 (15.2%)	40 (20.2%)	
- Mild Involvement	45 (45.5%)	46 (46.5%)	91 (46.0%)	
- Moderate Involvement	28 (28.3%)	38 (38.4%)	66 (33.3%)	
- Severe Involvement	1 (1.0%)	0 (0.0%)	1 (0.5%)	

**Table 3:** SINS total score.

Variable	Experimental (Mean ± SD)	Control (Mean ± SD)	Mean Difference (SE)	*p*-Value	Hedge’s g	95% CI
SINS	9.6 ± 2.8	10.5 ± 2.4	−0.9 (0.37)	0.018	−0.34	[−0.62, −0.06]

TOTAL SINS refers to Spinal Instability Neoplastic Score, pain categories and bone lesion classifications are based on clinical and radiological findings, p-values calculated using Pearson’s chi-squared test or ANOVA as appropriate.

**Table 4:** Comparison of Treatment Modalities Between Experimental and Control Groups.

Treatment Modality	Experimental (N = 99)	Control (N = 99)	Total (N = 198)	*p*-Value
Radiotherapy				<0.001
- No	5 (5.1%)	29 (29.3%)	34 (17.2%)	
- Yes	94 (94.9%)	70 (70.7%)	164 (82.8%)	
Chemotherapy				0.838
- None	23 (23.2%)	24 (24.2%)	47 (23.7%)	
- Standard Regimen	74 (74.7%)	74 (74.7%)	148 (74.7%)	
- Intensive Regimen	2 (2.0%)	1 (1.0%)	3 (1.5%)	
Surgical Treatment				0.022
- Missing Data	1 (1.0%)	1 (1.0%)	2 (1.0%)	
- No Surgery	74 (75.5%)	59 (60.2%)	133 (67.9%)	
- Surgery Performed	24 (24.5%)	39 (39.8%)	63 (32.1%)	

**Table 5:** Analysis of Survival Time and Complications in Experimental and Control Groups.

Variable	Experimental (N = 99)	Control (N = 99)	Total (N = 198)	*p*-Value
Survival Time from Diagnosis (Years)				<0.001
- Mean (SD)	3.5 (1.3)	2.8 (1.1)	3.1 (1.3)	
- Range	0.0–7.0	0.0–5.0	0.0–7.0	
Complications: Fractures				<0.001
- No Fractures	78 (79.6%)	43 (43.4%)	121 (61.4%)	
- Fractures Present	20 (20.4%)	56 (56.6%)	76 (38.6%)	
Complications: Osteoporosis				0.001
- No Osteoporosis	81 (82.7%)	61 (62.2%)	142 (72.4%)	
- Osteoporosis Present	17 (17.3%)	37 (37.8%)	54 (27.6%)	
Complications: Anemia				<0.001
- No Anemia	75 (77.3%)	43 (43.4%)	118 (60.2%)	
- Anemia Present	22 (22.7%)	56 (56.6%)	78 (39.8%)	
Complications: Vomiting				<0.001
- No Vomiting	91 (93.8%)	46 (46.5%)	137 (69.9%)	
- Vomiting Present	6 (6.2%)	53 (53.5%)	59 (30.1%)	
Pathological Fractures (Spine, Upper, Lower Limbs)				<0.001
- Mean (SD)	0.2 (0.4)	0.6 (0.5)	0.4 (0.5)	
- Range	0.0–2.3	0.0–1.0	0.0–2.3	
Pathological Fractures (Spine)				0.022
- No Fractures	81 (81.8%)	67 (67.7%)	148 (74.7%)	
- Fractures Present	18 (18.2%)	32 (32.3%)	50 (25.3%)	
Pathological Fractures (Upper Limbs)				0.070
- No Fractures	92 (92.9%)	84 (84.8%)	176 (88.9%)	
- Fractures Present	7 (7.1%)	15 (15.2%)	22 (11.1%)	
Pathological Fractures (Lower Limbs)				0.074
- No Fractures	91 (92.9%)	84 (84.8%)	175 (88.8%)	
- Fractures Present	7 (7.1%)	15 (15.2%)	22 (11.2%)	

**Table 6:** Comparison of Quality-of-Life Dimensions (EUROQOL) Between Experimental and Control Groups.

Dimension	Severity	Experimental (N = 99)	Control (N = 99)	Total (N = 198)	*p*-Value
Mobility	Minimal limitations	14 (14.1%)	0 (0.0%)	14 (7.1%)	<0.001
	Moderate limitations	55 (55.6%)	49 (49.5%)	104 (52.5%)	
	Severe limitations	30 (30.3%)	50 (50.5%)	80 (40.4%)	
Self-Care	Minimal limitations	12 (12.1%)	1 (1.0%)	13 (6.6%)	0.006
	Moderate limitations	62 (62.6%)	67 (67.7%)	129 (65.2%)	
	Severe limitations	25 (25.3%)	31 (31.3%)	56 (28.3%)	
Usual Activities	Minimal interference	4 (4.0%)	0 (0.0%)	4 (2.0%)	0.004
	Moderate limitations	72 (72.7%)	57 (57.6%)	129 (65.2%)	
	Severe limitations	23 (23.2%)	42 (42.4%)	65 (32.8%)	
Pain/Discomfort	Minimal pain/discomfort	1 (1.0%)	0 (0.0%)	1 (0.5%)	0.446
	Moderate pain/discomfort	48 (48.5%)	43 (43.4%)	91 (46.0%)	
	Severe pain/discomfort	50 (50.5%)	56 (56.6%)	106 (53.5%)	
Anxiety/Depression	Minimal anxiety/depression	10 (10.1%)	0 (0.0%)	10 (5.1%)	<0.001
	Moderate anxiety/depression	77 (77.8%)	59 (59.6%)	136 (68.7%)	
	Severe anxiety/depression	12 (12.1%)	40 (40.4%)	52 (26.3%)	

**Table 7:** Helplessness Depression Symptom Questionnaire Scores.

Variable	Experimental (Mean ± SD)	Control (Mean ± SD)	Mean Difference (SE)	*p*-Value	Hedge’s g	95% CI
HDSQ	52.1 ± 17.8	63.5 ± 12.9	−11.4 (2.21)	<0.001	−0.73	[−1.02, −0.44]

## Data Availability

The data supporting this study are available upon reasonable request from the corresponding author.
